# Ten-eleven translocation methyl-cytosine dioxygenase 2 deficiency exacerbates renal ischemia-reperfusion injury

**DOI:** 10.1186/s13148-020-00892-8

**Published:** 2020-07-02

**Authors:** Huan Yan, Li Tan, Yuqi Liu, Ning Huang, Jing Cang, Hao Wang

**Affiliations:** 1 Department of Anesthesiology, Zhongshan Hospital, Fudan University, Shanghai, 200032 People’s Republic of China; 2 Key Laboratory of Medical Epigenetics and Metabolism, Institutes of Biomedical Sciences, Fudan University, Shanghai, 200032 People’s Republic of China; 3 Department of Anesthesiology, Obstetrics and Gynecology Hospital, Fudan University, Shanghai, 200011 People’s Republic of China

**Keywords:** Tet2, Kidney, Cell junctions, Ischemia-reperfusion injury, Inflammatory response

## Abstract

**Background:**

Ten-eleven translocation (Tet) methyl-cytosine dioxygenases (including Tet1/2/3)-mediated 5mC oxidation and DNA demethylation play important roles in embryonic development and adult tissue homeostasis. The expression of *Tet2* and *Tet3* genes are relatively abundant in the adult murine kidneys while *Tet1* gene is expressed at a low level. Although Tet3 has been shown to suppress kidney fibrosis, the role of Tet2 in kidney physiology as well as renal ischemia-reperfusion (IR) injury is still largely unknown.

**Results:**

*Tet2*^*−/−*^ mice displayed normal kidney morphology and renal function as WT mice while the expression of genes associated with tight junction and adherens junction was impaired. At 24 h post-renal IR, *Tet2*^*−/−*^ mice showed higher SCr and BUN levels, more severe tubular damage, and elevated expression of *Kim1* and *Ngal* genes in the kidney in comparison with WT mice. Moreover, the transcriptomic analysis revealed augmented inflammatory response in the kidneys of *Tet2*^*−/−*^ mice.

**Conclusions:**

Tet2 is dispensable for kidney development and function at baseline condition while protects against renal IR injury possibly through repressing inflammatory response. Our findings suggest that Tet2 may be a potential target for the intervention of IR-induced acute kidney injury (AKI).

## Introduction

Ten-eleven translocation (Tet) family methyl-cytosine dioxygenases (Tet1, Tet2, and Tet3) are key enzymes to convert 5-methylcytosine (5mC) to 5-hydroxymethylcytosine (5hmC), 5-formylcytosine (5fC), and 5-carboxylcytosine (5caC) [1–3]. Tet protein-catalyzed 5mC oxidation generates new epigenetic modifications and initiates active and passive DNA demethylation [4]. Emerging evidence has shown that *Tet* family genes and 5mC oxidation are critical for not only embryonic development but also the maintenance of adult tissue homeostasis [5]. Tet1–3 have differential expression pattern in the kidney, with relatively high expression of Tet2 and Tet3 and low expression of Tet1 [6, 7]. We have previously shown that *Tet2* is dramatically downregulated in the IR-injured kidney, which is accompanied by a reduction of the global 5hmC level [7]. By performing hydroxymethylated DNA immunoprecipitation sequencing (hMeDIP-seq) analysis, we also revealed that 5hmC was enriched in the gene body regions of renal IR injury-associated genes [8]. In addition, a few studies have shown that Tet3 confers protection against kidney fibrosis [9–11].

These above-mentioned studies suggest that *Tet* family genes as well as 5mC oxidation may play important roles in regulating the physiological and pathophysiological processes in the kidney. However, the functional importance of *Tet2* gene in the kidney as well as renal IR injury remains unknown. Using a unique *Tet2*^*−/−*^ mouse model, here, we determined the impact of Tet2 deficiency on the function of the kidney at baseline as well as in response to renal IR injury. Our findings suggest that Tet2 is dispensable for kidney development but limits kidney damage following IR. Mechanistically, we identified that Tet2 regulates a specific set of genes that are involved in cell junctions and inflammatory response. Thus, our findings provide new insights into the molecular mechanism regulating kidney homeostasis and renal IR injury.

## Results

### Tet2 deficiency does not influence kidney development and normal renal function

**Fig. 1:**
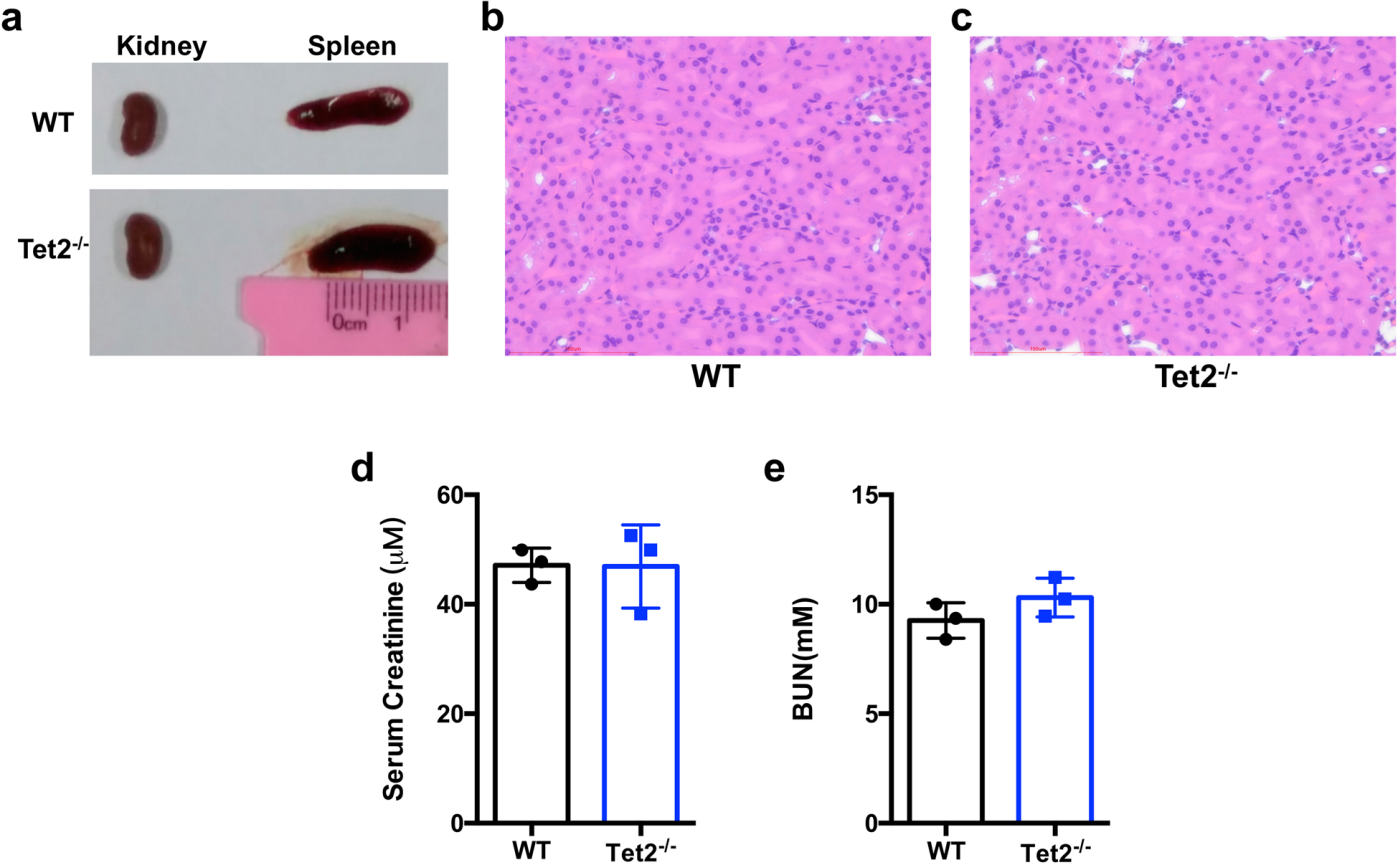
Tet2 deficiency does not influence kidney development and normal renal function. **a** Morphology of the kidneys and spleens from WT and *Tet2*^*−/−*^ mice at 6 weeks old. **b**, **c** H&E staining of the kidneys from WT and *Tet2*^*−/−*^ mice at 6 weeks old. **d**, **e** Serum creatinine (**d**) and blood urea nitrogen (**e**) concentrations of WT and *Tet2*^*−/−*^ mice under baseline conditions. *N* = 3 in each group

*Tet2*^−/−^ mice could spontaneously develop chronic myelomonocytic leukemia (CMML) in their adult stage (starting from 2–4 months) [12–15]. To avoid the secondary effect of Tet2 depletion-induced hematopoietic dysfunction on the kidney, we used 6-week-old male mice in the current study. Indeed, there were no CMML-like pathological changes in the peripheral blood, spleen, and bone marrow of *Tet2*^*−/−*^ mice at a young age (data not shown). Importantly, there was no difference in the morphology and histological structure of kidneys between WT and *Tet2*^*−/−*^ groups of mice (Fig. 1a–c). Moreover, the SCr and BUN levels (two plasma markers of renal dysfunction) in *Tet2*^*−/−*^ mice were also comparably low as those in WT mice (Fig. 1d, e).

### Tet2 deficiency impairs the expression of genes associated with cell junctions in the kidney

**Fig. 2:**
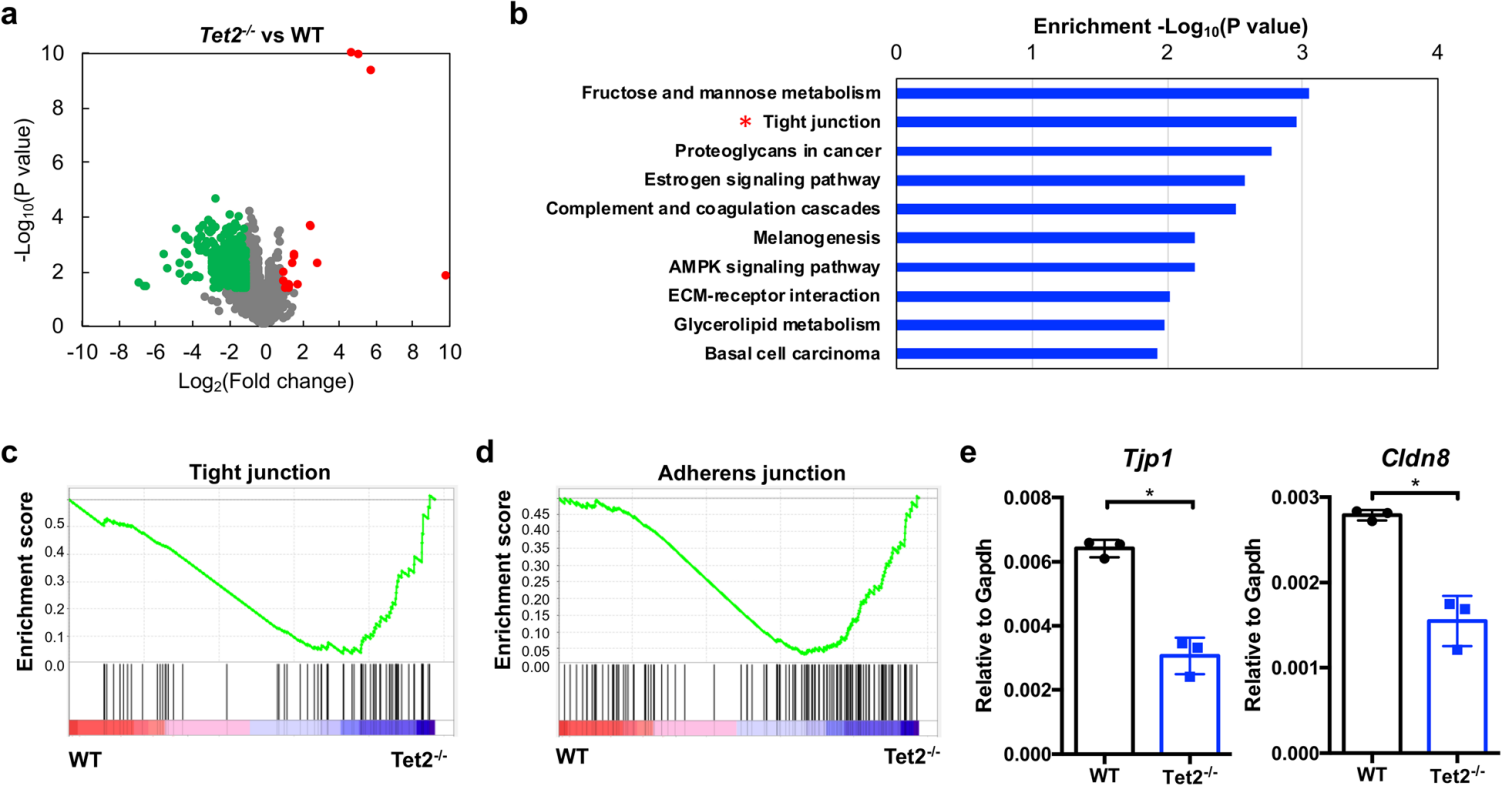
Tet2 deficiency impairs the expression of genes associated with cell junctions in the kidney. The kidneys from WT and *Tet2*^*−/−*^ mice (*n* = 3 in each genotype) were subjected to RNA-seq analysis. **a** Scatter plotting of the differentially expressed genes in the kidneys between WT and *Tet2*^*−/−*^ mice. Green dot, downregulated gene; red dot, upregulated gene. **b** Top 10 terms of the pathway analysis of the downregulated genes. **c**, **d** Gene set enrichment analyses (GSEA) of RNA-seq data for WT and *Tet2*^*−/−*^ mouse kidneys, revealing the association of the gene program in *Tet2*^*−/−*^ mouse kidneys with the “tight junction” (**c**) and “adherens junction” (**d**) gene signature. **e** qRT-PCR analysis of two representative genes (*Tjp1* and *Cldn8*) in the kidneys from WT and *Tet2*^*−/−*^ mice. *P < 0.05. *N* = 3 in each group

To understand the role of Tet2 in the kidney, we performed RNA-seq analysis of the transcriptomes in WT and *Tet2*^*−/−*^ mice kidneys. Compared with WT mice, 452 differentially expressed genes (24 upregulated genes and 428 downregulated genes) were identified in the kidneys of *Tet2*^*−/−*^ mice (Fig. 2a). KEGG pathway analysis revealed that “fructose and mannose metabolism,” “tight junction,” and “proteoglycans in cancer” were the top three enriched terms in the downregulated genes (Fig. 2b). Gene set enrichment analyses (GSEA) revealed that the downregulated genes in the kidney tissues of *Tet2*^*−/−*^ mice were significantly enriched for the “adherens junction” and “tight junction” gene sets (Fig. 2c, d). Moreover, key regulators in the above biological processes, i.e., *Cldn8* and *Tjp1*, were validated by RT-qPCR (Fig. 2e).

### Tet2 deficiency exacerbates renal IR injury in mice

**Fig. 3:**
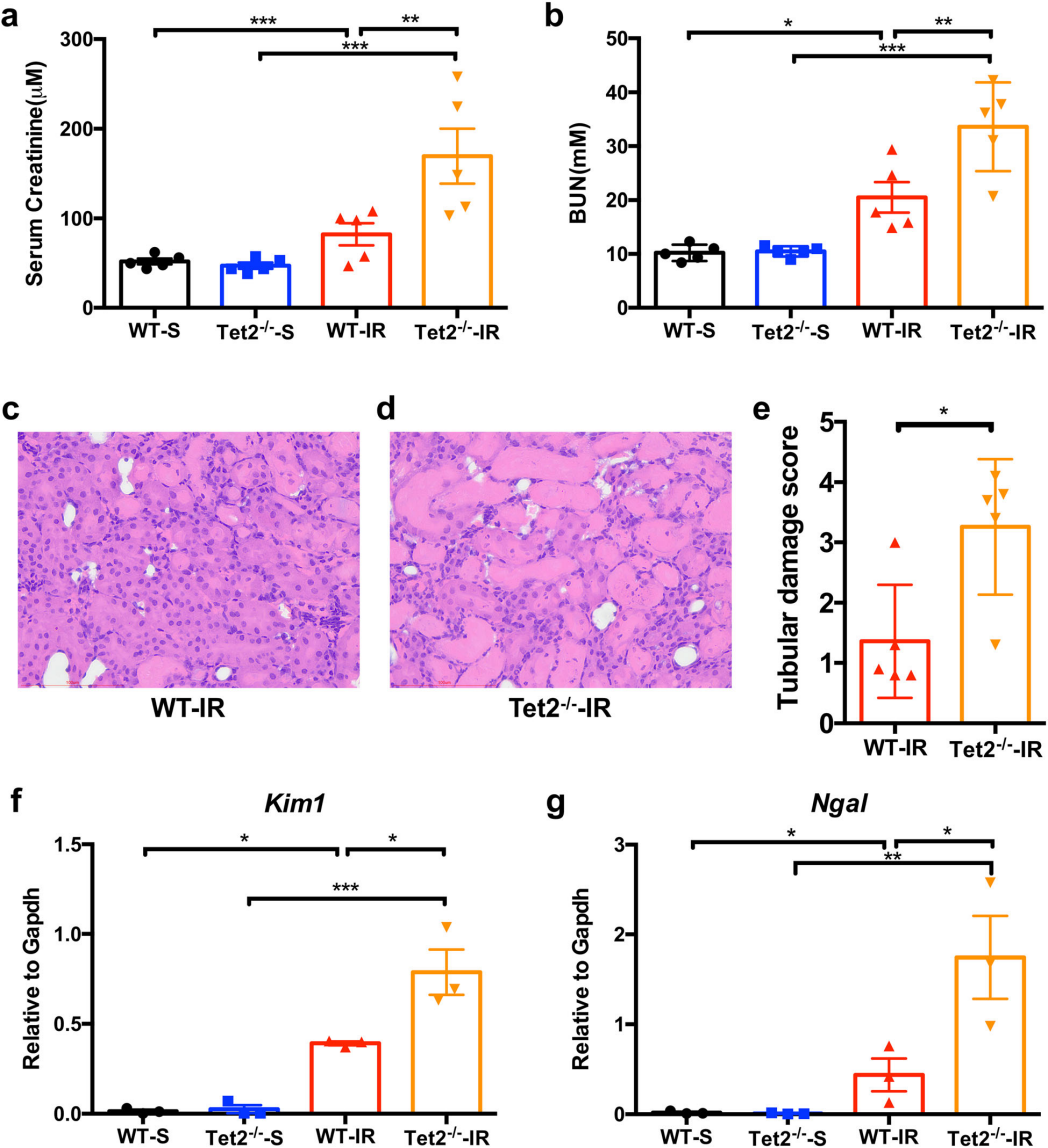
Tet2 deficiency exacerbates renal IR injury in mice. Mice were subjected to sham operation or 60 min of warm kidney ischemia. Blood and kidney tissues were collected at 24 h after reperfusion for the following analyses. S, sham-operated; IR, ischemia-reperfusion injury; WT, wild type; *Tet2*^*−/−*^, *Tet2* knockout mice. **a**, **b** Serum creatinine (**a**) and blood urea nitrogen (**b**) concentrations of WT and KO mice. *P < 0.05, **P < 0.01, and ***P < 0.001. *N* = 5 in each group. **c**, **d** Representative micrographs showing the morphology of the IR-insulted kidneys from WT (**c**) and *Tet2*^*−/−*^ (**d**) mice. Arrows show the injury. **e** Quantitative assessment of tubular damage. *P < 0.05. *N* = 5 in each group. **f**, **g** The mRNA levels of *Kim1* and *Ngal* were measured by qRT-PCR. *P < 0.05, **P < 0.01, and ***P < 0.001. *N* = 3 in each group

We then examined whether Tet2 depletion affects the response of the kidney to pathological stimulus. WT and *Tet2*^*−/−*^ mice were subjected to sham operation or renal IR. Compared with the sham group, IR significantly increased SCr and BUN levels in WT mice, both of which were further elevated in *Tet2*^*−/−*^ mice in response to renal IR (Fig. 3a, b). As revealed by H&E staining, IR caused tubular detachment, foamy degeneration, and necrosis in the kidneys from both genotypes of mice (Fig. 3c, d). However, *Tet2*^*−/−*^ mice displayed more severe tubular damage than WT mice (Fig. 3c–e). Moreover, the mRNA levels of *Kim1* and *Ngal*, two well-known renal IR injury marker genes [16, 17], were significantly higher in the IR-insulted kidneys from *Tet2*^*−/−*^ mice than those from WT mice (Fig. 3f, g).

### Tet2 deficiency boosts inflammation in kidney upon IR injury

**Fig. 4:**
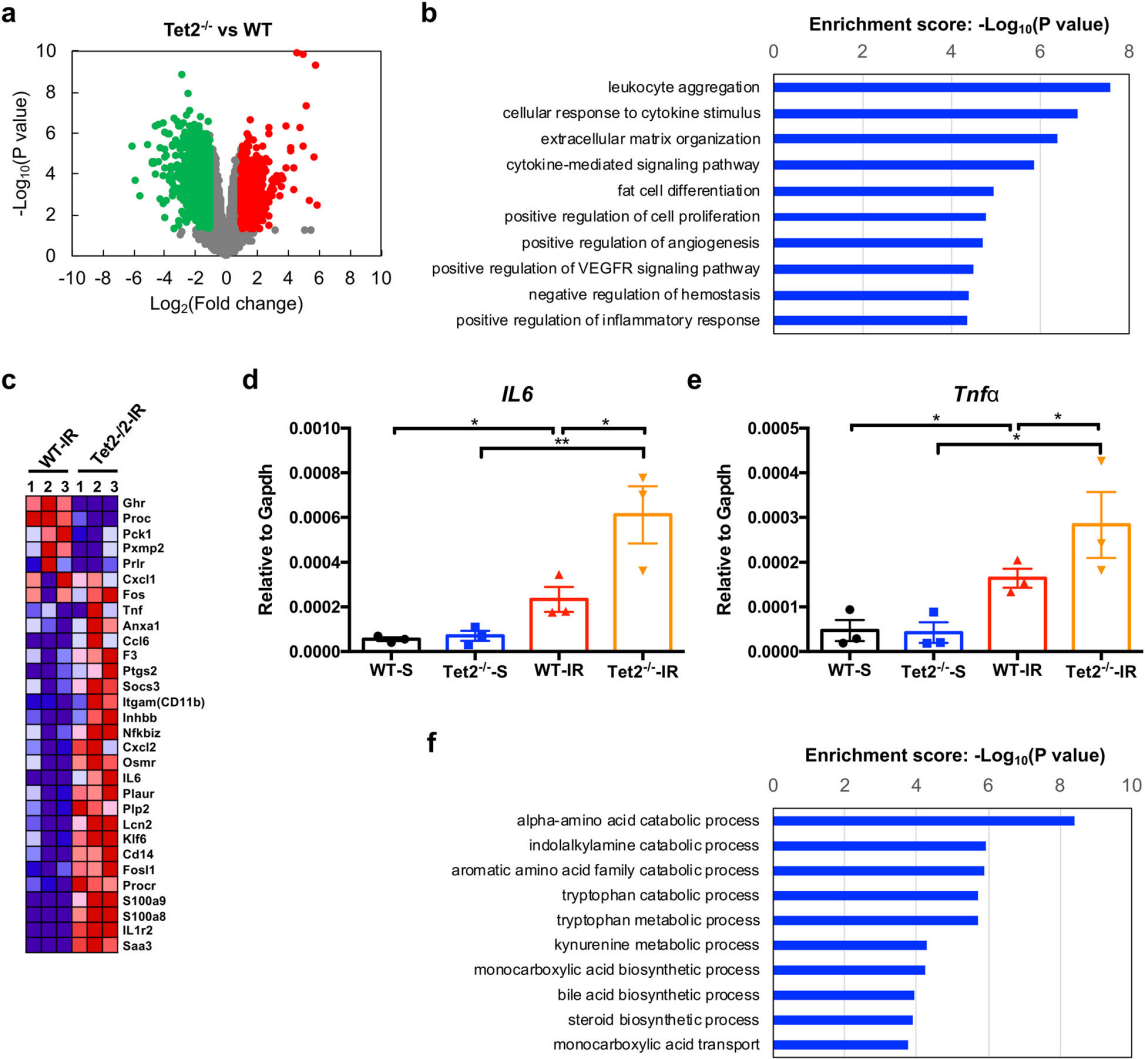
Tet2 deficiency boosts inflammation in the kidney upon IR injury. The IR-insulted kidney tissues from WT and *Tet2*^*−/−*^ mice (*n* = 3 in each genotype) were subjected to RNA-seq analysis. **a** Scatter plotting of the differentially expressed genes in the IR-insulted kidney tissues from WT and *Tet2*^*−/−*^ mice. Green dot, downregulated genes; red dot, upregulated genes. **b** Top 10 terms of the pathway analysis of the upregulated DEGs in the IR-insulted kidney between WT and *Tet2*^*−/−*^ mice. **c** Heatmap showing the augmented expression of renal inflammation-related genes. **d**, **e** The mRNA levels of *IL6* (**d**) and *TNF-alpha* (**e**) were measured by qRT-PCR. *P < 0.05 and **P < 0.01. *N* = 3 in each group. **f** Top 10 terms in the pathway analysis of downregulated DEGs in the IR-insulted kidney between WT and *Tet2*^*−/−*^ mice

To determine the potential molecular mechanism underlying the protective role of Tet2 in the kidney, we profiled the transcriptomes of the IR-insulted kidneys from WT and *Tet2*^*−/−*^ mice. Compared to the counterparts from WT mice, the IR-insulted kidneys from *Tet2*^*−/−*^ mice displayed 928 upregulated genes and 1092 downregulated genes (Fig. 4a). KEGG pathway analyses for the upregulated genes identified “leukocyte aggregation,” “cellular response to cytokine stimulus,” “negative regulation of hemostasis,” and “positive regulation of inflammatory response” in the top 10 enriched terms (Fig. 4b). Persisting inflammation contributes to renal damage following IR. We found that the mRNA levels of pro-inflammatory genes (*Il6*, *Tnf-alpha*, and *Il1-beta*) and chemokine genes (*Cxcl2*, *Ccl6*, and *Ccl2*) are significantly higher in the IR-insulted kidneys from *Tet2*^*−/−*^ mice than those from WT mice (Fig. 4c). The changes in *Il6* and *Tnf-alpha* mRNAs were validated by qRT-PCR (Fig 4d, e). Consistently, higher expression levels of macrophage markers (*CD14* and *CD11b*) and neutrophil markers (*S100a8* and *S100a9*) were also observed in the IR-insulted kidneys from *Tet2*^*−/−*^ mice (Fig. 4c). For the downregulated genes, metabolism processes (such as catabolic process, metabolic process, and biosynthetic process) were enriched (Fig. 4f), suggesting that Tet2 deficiency may dampen the renal metabolism during IR injury.

## Discussion

In the current study, we used WT and *Tet2*^*−/−*^ mice as a comparative model to explore the functional role of Tet2 in the kidney and demonstrated that Tet2 deficiency impairs the expression of genes associated with cell junctions and exacerbates IR-induced AKI. These findings extended our understanding of the physiological and pathophysiological function of Tet2, a key regulator of DNA demethylation. To our knowledge, this work for the first time establishes the functional role of Tet2 in the kidney.

Since Rao and colleagues identified TET1 as the first 5mC dioxygenase in 2009, *TET* family genes have attracted increasing attention regarding their physiological function in various contexts [1, 4]. Our data uncover that *Tet2* gene is dispensable for kidney development and physiological function as its deficiency does not alter the morphology, histological structure, or functional markers of mouse kidney. This observation is consistent with the results of previous studies that Tet2 knockout mice had no significant phenotype despite hematological lineage dysregulation in the adult age [12, 14, 18]. While the loss of Tet2 does not impair kidney development and function, our transcriptomic analysis revealed that a panel of genes associated with cell junctions were inhibited in the kidneys of *Tet2*^*−/−*^ mice. Cell junctions (including tight junction (TJ), adherens junction (AJ), gap junction (GJ), desmosomes, and hemidesmosomes) play important roles in the epithelial homeostasis [19, 20]. For instance, tight junctions at the most apical part of the basolateral plasma membrane create a selective diffusion barrier for epithelium [21]. Consistently, a recent study has shown that Tet2 depletion increases the intestinal permeability (especially the jejunum), leading to microbial leaky and chronic inflammation, which function as an extrinsic non-cell-autonomous factor for leukemia onset [22]. Therefore, the aberrant expression of cell junction genes indicates that Tet2 depletion may influence kidney function under stress conditions.

Our previous work has shown that *Tet2* gene expression was downregulated in murine kidneys insulted by IR injury [7]. Therefore, we evaluated the impact of Tet2 deficiency on renal IR injury in this study and uncovered that *Tet2* deficiency increases the susceptibility to renal IR injury. Miao et al. have reported that *Tet2* knockout worsened ischemic brain injury in a middle cerebral artery occlusion (MCAO) mouse model [23]. Their work together with ours indicates that Tet2 plays a protective role against IR injury and that such effect is not limited to a specific organ.

During the renal IR injury, oxidative stress induces necrosis of endothelial or epithelial cells, leading to sterile inflammation and amplified acute kidney injury [24]. Consistent with more severe AKI, we observed much higher expression levels of pro-inflammatory genes and immune cell markers in the IR-insulted kidneys from *Tet2*^*−/−*^ mice. It has been reported that Tet2 knockout macrophages were more sensitive to lipopolysaccharide-induced IL6 production [25]. However, it is unclear whether such a stronger immune response in the IR-insulted kidneys of *Tet2*^*−/−*^ mice is due to a direct effect of Tet2 loss on the immune system or rather a consequence of more severe renal tubule injury.

As one of the key epigenetic regulators, TET2 has 5mC dioxygenase activity and exerts its function in regulating chromatin structure and gene transcription through two mechanisms [4]. First, TET2 can convert 5mC to 5hmC/5fC/5caC and thereby initiate passive or active DNA demethylation. TET2 deficiency may impair gene transcription due to aberrant DNA hypermethylation on the regulatory regions such as promoters and enhancers [26, 27]. Alternatively, TET2 functions as a transcriptional co-repressor through recruiting SIN3A and HDAC1/2 histone deacetylases to chromatin and triggers histone deacetylation, which in turn inhibits gene transcription [28]. Given that both up- and downregulated genes were observed in the kidneys of *Tet2*^*−/−*^ mice compared to WT mice, our findings indicate that both epigenetic regulatory mechanisms are likely to be involved in the renal protective role of TET2.

As one of the hotspots mutated genes in the peripheral blood mononuclear cells (PMBCs) of the elderly population, which was also known as clonal hematopoiesis of indeterminate potential (CHIP) [29–31], *TET2* mutation is associated with an increased risk for atherosclerosis and cardiac diseases [32]. However, the impact of hematopoietic TET2 mutation on the risk of acute or chronic kidney diseases in elder people is still unclear. In general, old patients are more vulnerable to IR-induced AKI than young ones [33]. Given that 5hmC (an index of TET activity) level was reduced in the kidney during aging [34], our findings indicate that the age-associated decline in TET activity may contribute to the increased risk of AKI and chronic kidney diseases in elderly patients. Since recent studies have shown that vitamin C could work as a direct regulator of Tet activity in embryonic stem cells (ESCs) and hematopoietic stem cells (HSCs) [35, 36], it will be of great interest to determine whether vitamin C exerts its substantial clinical benefits on renal IR injury partially through activation of Tet2.

## Conclusion

Taken together, our study demonstrates that *Tet2* deficiency impairs the expression of genes associated with cell junctions in mouse kidney and increases the susceptibility to renal IR injury. These findings highlight a protective role of Tet2 in the kidney against IR-induced AKI and indicate that Tet2 may be a potential target for the prevention and therapy of perioperative AKI.

## Methods

### Animals

Breeding pairs of *Tet2*^*+/−*^ mice with a C57BL/6J background were kindly provided by Dr. Guoliang Xu (State Key Laboratory of Molecular Biology, Institute of Biochemistry and Cell Biology, Chinese Academy of Sciences) [37]. Wild-type (WT) and *Tet2*^*−/−*^ (KO) mice were generated according to the half-sib mating and housed under specific pathogen-free conditions and a 12 h/12 h light/dark cycle at 21–23 °C with ad libitum food and water. A total of 26 male mice (13 WT and 13 *Tet2*^*−/−*^) at a young age (6 weeks old) were used in the current study. Three pairs of mice were used for baseline condition analysis, and ten pairs of mice were used for renal IR injury as well as sham operation. All experiments were approved by the Institutional Animal Care and Use Committee of Zhongshan Hospital, Fudan University (protocol no. 20170207), and conformed to the Animal Research-Reporting In Vivo Experiments (ARRIVE) guidelines.

### Renal IR injury

WT and *Tet2*^*−/−*^ male mice at around 6 weeks old were subjected to renal IR injury or sham operation. The renal IR injury model was performed as described previously [38]. In brief, mice were anesthetized with isoflurane. After an abdominal incision was made, IR injury was induced by clamping the renal pedicle of the right kidney for 60 min. After the clamps were released, the left kidney was removed. Before the wounds were sutured, 2 ml of saline was infused into the peritoneal cavity to keep the animals well hydrated. During the procedure, mice were warmed on a heating pad (36 °C) to maintain their body temperature. The mice under sham operation experienced a similar procedure except for renal ischemia. At the end of the experiment (24 h post-operation), all animals were euthanized by overdose (100 mg kg^−1^) of sodium pentobarbital and the blood and tissues were collected for the analyses of serum creatinine (SCr) and blood urea nitrogen (BUN), RNA-seq, qRT-PCR, and histology.

### RNA analysis

Total RNA was isolated from the kidney using TRIzol reagent (Life Technologies) according to the manufacturer’s instructions.

#### RNA-seq

RNA-seq library was constructed and sequenced by WuXi NextCODE (Shanghai). RNA-seq reads were aligned to the University of California Santa Cruz (UCSC) mouse genome mm9 using STAR. The tag directories were established. The raw read counts were measured with RefSeq genes using Homer, and the differential gene expression analysis was carried out using edgeR. The differential expression genes (DEGs) between WT and *Tet2*^*−/−*^ kidneys were defined by over 1 or − 1 in the difference FPKM log_2_ value and FDR *P* value less than 0.05. Gene ontology (GO) and KEGG pathway analyses for DEGs were performed using DAVID functional annotation tools. Gene set enrichment analyses (GSEA) were performed according to the instructions. The raw RNA-seq data have been deposited in NCBI’s GEO (Gene Expression Omnibus) and are accessible through the GEO Series accession number GSE151260.

#### RT-qPCR

Reverse transcription (RT) of cDNA was performed using the PrimeScript RT reagent Kit with gDNA Eraser (TaKaRa). Quantitative polymerase chain reaction (PCR) was conducted with SYBR Premix Ex Taq (TaKaRa) on the Applied Biosystems 7500 Real-Time PCR System. Levels of Gapdh were used as an internal control for the normalization of RNA quantity and quality differences among the samples. The sequence information of PCR primers is shown in the supplementary data (Table 1).

**Table 1:** The sequences of qRT-PCR primers

GeneBank ID	Gene symbol	Primer sequence (forward/reverse)
NM_008084	Gapdh	5′-GTGTTCCTACCCCCAATGTGT-3′ 5′-ATTGTCATACCAGGAAATGAGCTT-3′
NM_009386	Tjp1 (ZO-1)	5′-CCACCTCTGTCCAGCTCTTC-3′ 5′-CACCGGAGTGATGGTTTTCT-3′
NM_018778	Cldn8	5′-TCCCAAGGCGTACAGATTTC-3′ 5′-CACTCTCCACTGAGGCATGA-3′
NM_134248	Kim1 (Havcr1)	5′-AGCTCAGGGTCTCCTTCACA-3′ 5′-ACCACCCCCTTTACTTCCAC-3′
NM_008491	Ngal (Lcn2)	5′-CTGAATGGGTGGTGAGTGTG-3′ 5′-GGAGTGCTGGCCAAATAAGA-3′
NM_031168	Il-6	5′-AGTTGCCTTCTTGGGACTGA-3′ 5′-TCCACGATTTCCCAGAGAAC-3′
NM_013693	Tnf-alpha	5′-GAAAAGCAAGCAGCCAACCA-3′ 5′-CGGATCATGCTTTCTGTGCTC-3′

### Assessment of renal function

Blood was centrifuged to collect serum. Serum creatinine (SCr) and blood urea nitrogen (BUN) were measured as markers of renal function using Creatinine Assay Kit (QuantiChrom, DICT-500) and Urea Assay Kit (QuantiChro, DIUR-500).

### Histology

Formalin-fixed kidney sections were cut into 4 μm slides and stained with hematoxylin and eosin (H&E). The percentage of tubules in the cortical-medullary junction that displayed cellular necrosis, loss of brush border, cast formation, and tubular dilatation was counted and scored in a blinded manner as follows: 0, none; 1, 0–10%; 2, 11–25%; 3, 26–45%; 4, 46–75%; and 5, > 75%. At least 10 high-power fields (HPFs, × 200 magnification) per section for each sample were examined. The pathologists who scored the degree of the injury were blinded.

### Statistical analysis

GraphPad Prism 6 (GraphPad, La Jolla, CA, USA) was used for statistical analyses and graphs. Data are expressed as mean ± SEM (standard error of the mean). An unpaired *t* test was applied for two-group analyses. One-way ANOVA with Turkey’s test was applied for four-group analyses. For all analyses, *P* < 0.05 was considered significant.

## Data Availability

Based on a reasonable request, the data from the current research analysis can be obtained from the corresponding author. The raw RNA-seq data are available through the GEO Series accession code GSE151260.

## Abbreviations

Tet2: Ten-eleven translocation 2
5hmC: 5-Hydroxymethylcytosine
IR: Ischemia-reperfusion
AKI: Acute kidney injury
SCr: Serum creatinine
BUN: Blood urea nitrogen
CMML: Chronic myelomonocytic leukemia
TJ: Tight junction
AJ: Adherens junction
GJ: Gap junction
DEGs: Differential expression genes
CHIP: Clonal hematopoiesis of indeterminate potential
PMBCs: Peripheral blood mononuclear cells
